# A Case Report of Hydatid Cyst Mimicking Thyroid Mass With Mediastinal Extension and Bony Erosion: A Successful Non-surgical Approach to Treatment

**DOI:** 10.7759/cureus.101342

**Published:** 2026-01-12

**Authors:** Maham Ansari, Aun Raza, Waqas Shafiq, Salma Abbas, Ain-ul-Yaqeen M Malik

**Affiliations:** 1 Medicine, Shaukat Khanum Memorial Cancer Hospital and Research Centre, Lahore, PAK; 2 Infectious Diseases and Internal Medicine, Shaukat Khanum Memorial Cancer Hospital and Research Centre, Lahore, PAK; 3 Endocrinology and Diabetes, Shaukat Khanum Memorial Cancer Hospital and Research Centre, Lahore, PAK; 4 Histopathology, Shaukat Khanum Memorial Cancer Hospital and Research Centre, Lahore, PAK

**Keywords:** albendazole, bony destruction, cystic echinococcosis, hydatid disease, sternoclavicular joint

## Abstract

We report an unusual manifestation of hydatid disease, presenting as a progressively enlarging, painless swelling in the right infraclavicular region. Imaging studies revealed a large necrotic mass extending into the superior mediastinum with the destruction of the adjacent right sternoclavicular joint; this was initially suspected to be thyroidal in origin. However, histopathological examination was consistent with a ruptured hydatid cyst, and *Echinococcus* serology was positive. Due to anatomical considerations, surgical intervention was not pursued, and the patient was managed with oral anthelmintic therapy, resulting in partial radiological response after 12 weeks of therapy. This case underscores the importance of considering hydatid disease as a differential diagnosis, even with atypical anatomical involvement. It also highlights the potential role of medical management alone as a viable alternative when surgery is not feasible.

## Introduction

Hydatid disease or cystic echinococcosis (CE) is a parasitic zoonosis caused primarily by *Echinococcus granulosus*. *E. granulosus* sensu stricto is the predominant species causing human disease worldwide, with *E. canadensis* and *E. ortleppi* contributing to a minority of cases, differentiated via molecular methods [1]. Endemic areas include parts of South America, Africa, the Eastern Mediterranean, the Middle East, and Asia. In Pakistan, 1,611 cases of CE were recorded between 1990 and 2018 [2]. Transmission occurs via dogs as primary definitive hosts and different livestock species as intermediate hosts. Human infection results from the accidental ingestion of infective eggs of* E. granulosus* through contact with feces, fur of infected dogs, and contaminated water and food [1]. 

The liver (70%) and lungs (20%) are most commonly involved, with infrequent involvement of the brain, spleen, kidney, and heart [1]. Hydatid disease in the head and neck region is extremely rare, with only a few cases reported [3-5]. Extension into the thoracic cavity without pulmonary involvement is also unusual, with mediastinal involvement being reported in 4.5-14% of these cases [6,7]. Furthermore, among all the reported cases, bony erosion due to hydatid cyst is seldom seen [8], with CE of the skeletal system accounting for only 0.5-4% of cases, most commonly affecting the vertebrae (35-60%), followed by the pelvis, femur, ribs, humerus, tibia, scapula, and cranium [9]. 

## Case presentation

A 57-year-old lady from the north of Pakistan with diabetes and hypertension presented with a one-year history of slowly progressive, painless swelling in the right infraclavicular region, with dilated veins inferior to this area. No fever, weight loss, respiratory symptoms, or swallowing difficulty was noted. Her medical history was notable for neck surgery 25 years ago for a swelling deemed a "goiter". Additionally, she underwent liver surgery approximately 20 years ago; symptoms at that time included right upper quadrant pain and vomiting. Prior surgical records were unavailable. On clinical examination, the patient's vital signs were unremarkable. A transverse thyroidectomy scar was seen. A right infraclavicular, non-tender, cystic lesion measuring about 8×6 cm was noted, with minimal erythema of the overlying skin and dilated, tortuous veins over the chest wall inferior to this area. There was no facial edema or other cutaneous changes. 

Diagnosis and management

Complete blood count was unremarkable except for eosinophilia, and thyroid function tests were within range (Table 1). Initial ultrasonography revealed an 8×5 cm multicystic lesion with microcalcification, contiguous with the right thyroid lobe and protruding inferiorly into the retrosternal region.

**Table 1:** Laboratory findings The values/results in bold indicate findings which may be of particular interest to the reader/have bearing on the case, i.e., normal TSH and raised *Echinococcus* IgG and eosinophils. TSH: thyroid-stimulating hormone; ALT: alanine aminotransferase; AST: aspartate aminotransferase; GGT: gamma-glutamyl transferase; IgG: immunoglobulin G; DU: diagnostic units; WBC: white blood cell

Parameter	Result	Reference range
TSH	1.4 µIU/mL	0.4-4.0 µIU/mL
ALT	38 U/L	<34 U/L
AST	30.9 U/L	<31 U/L
GGT	166 U/L	10-54 U/L
Total bilirubin	0.43 mg/dL	0.3-1.0 mg/dL
Alkaline phosphatase	132.88 U/L	46-122 U/L
*Echinococcus* antibody IgG	25.255 DU	>11 DU (positive)
Creatinine	0.62 mg/dL	0.6-1.3 mg/dL
WBC	7.34x10³/µL	4.5-11x10³/µL
Hemoglobin	11.8 g/dL	11-14.4 g/dL
Platelets	204x10³/µL	150-450x10³/µL
Neutrophil	44.5%	40.7-70.7%
Lymphocyte	34.1%	20.2-40%
Monocyte	9%	3-10%
Eosinophil	11.9%	0.5-6%
Basophil	0.5%	0.2-1.1%

**Figure 1:**
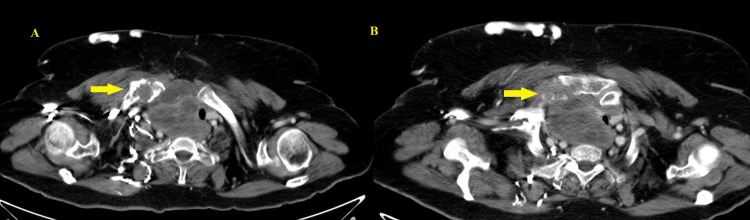
CT imaging from May 2025 A large predominantly cystic lesion was identified in the right lower neck with the involvement of the adjacent right thyroid gland as well. This shows heterogeneous osseous expansion with the involvement of the medial end of the clavicle (A) and right sternoclavicular joint with its erosion (B). CT: computed tomography

Computed tomography (CT) imaging revealed a large necrotic neck mass, inseparable from the right lobe of the thyroid, with the bulk within the superior mediastinum. It extended to involve the right sternoclavicular joint, with its destruction (Figure 1). Significant mass effect on the trachea and esophagus with contralateral shift and tracheal luminal narrowing and near-complete occlusion of the superior vena cava without direct invasion was seen. Calcific changes were seen within the liver segments 7 and 5. A magnetic resonance imaging (MRI) scan was performed, with similar findings.

**Figure 2:**
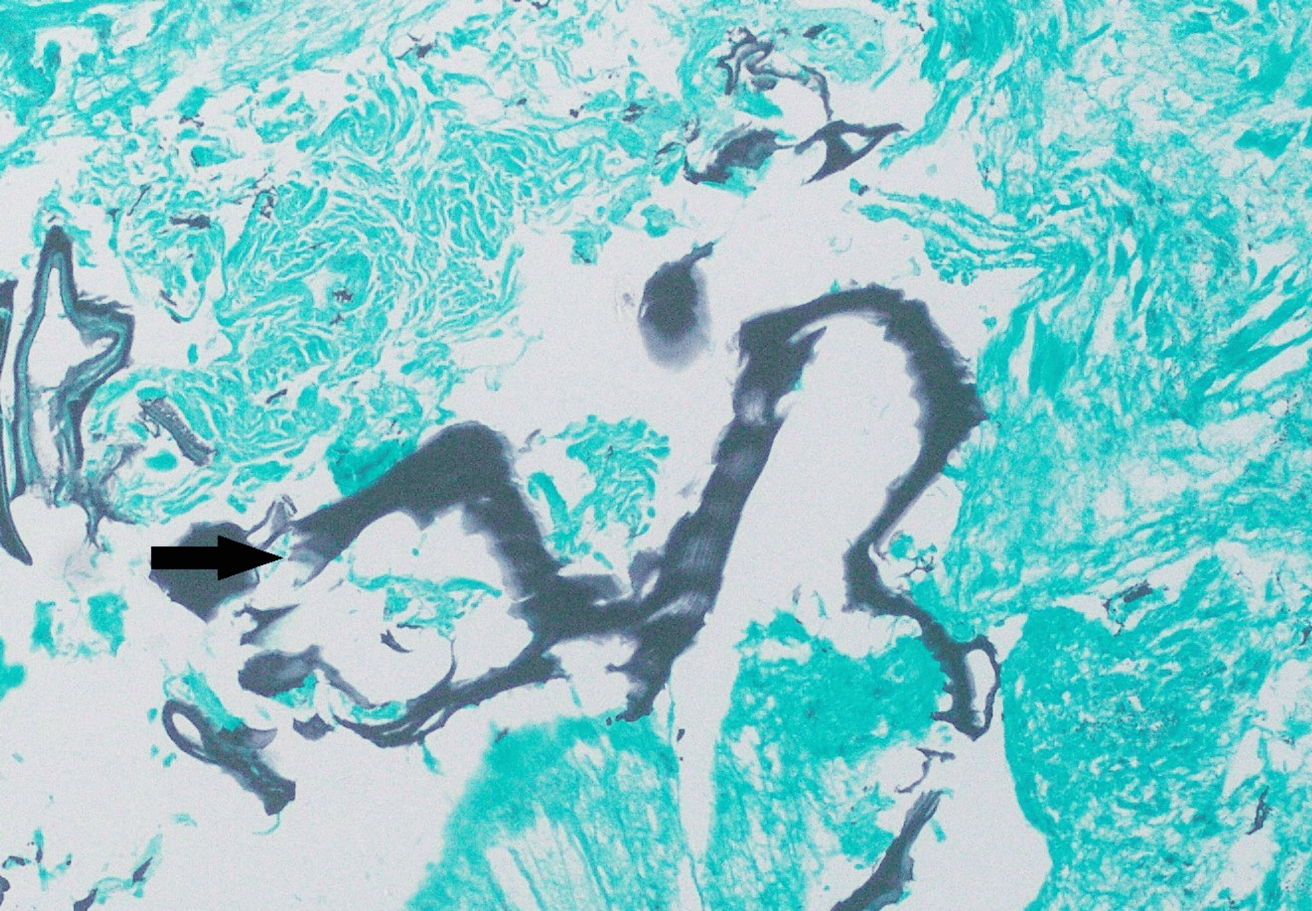
Grocott-Gomori's methenamine silver stain highlighting the laminated membrane (black arrow)

**Figure 3:**
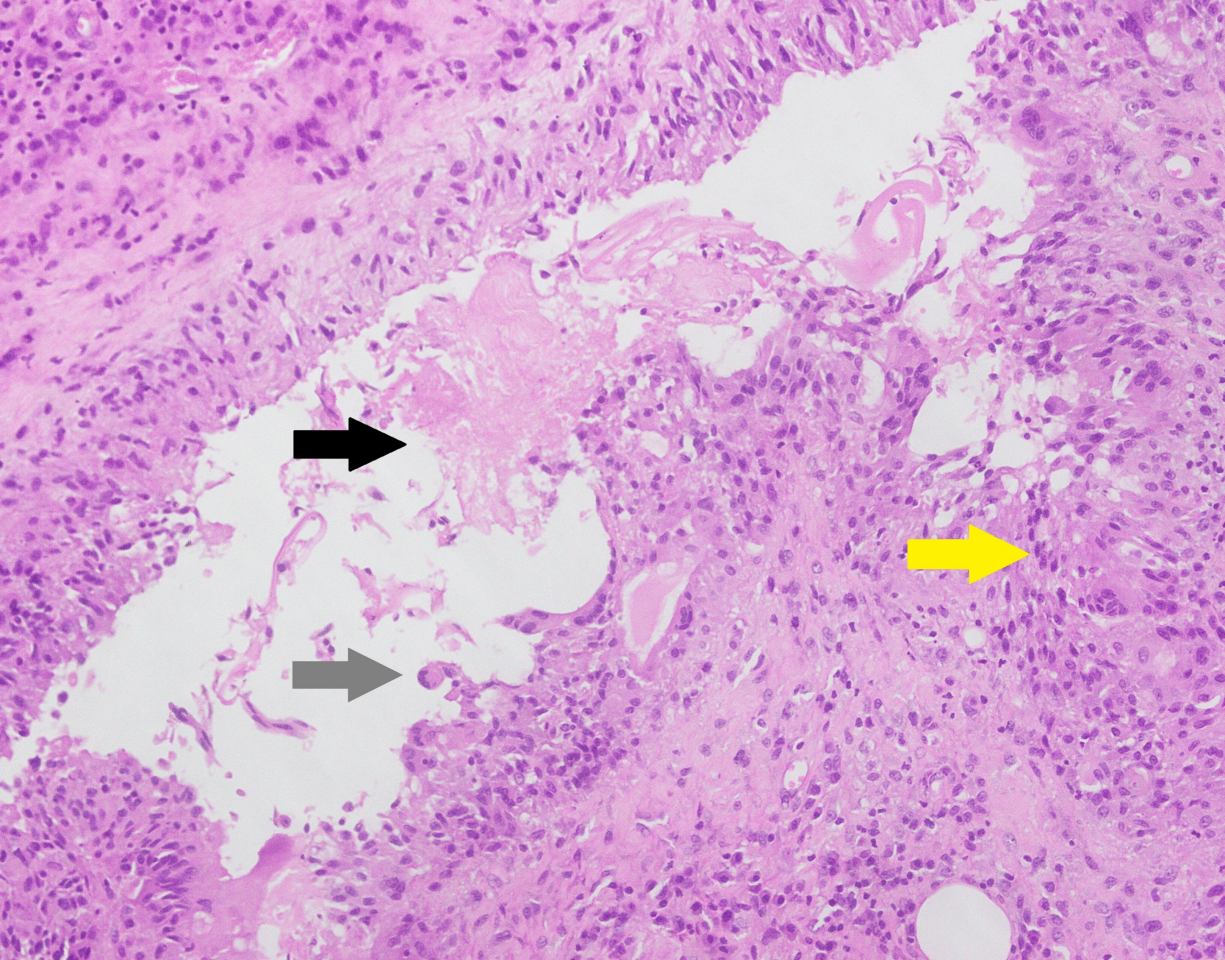
H&E stain of the biopsy sample Laminated membrane-like material (black arrow), granulomatous response comprising epithelioid histiocytes and foreign body giant cells (yellow arrow), and foreign body giant cell (grey arrow). H&E: hematoxylin and eosin

Fine-needle aspiration (FNA) biopsy performed prior to visiting our hospital revealed skeletal muscle with inflammation and foreign body giant cell reaction. This was deemed non-representative of the whole mass, and an incisional biopsy was performed, which revealed granulomatous reaction with foreign body giant cells and a laminated membrane, suggestive of a ruptured hydatid cyst; no protoscolices or germinal membrane was identified (Figure 2, Figure 3).

She was referred to the Infectious Diseases clinic; her detailed history did not reveal any contact with dogs or cattle. She lived in a rural area but did not have any significant animal husbandry exposure. Based on her surgical history and biopsy findings, *Echinococcus* serology was performed using enzyme-linked immunosorbent assay (ELISA) and returned positive.

**Figure 4:**
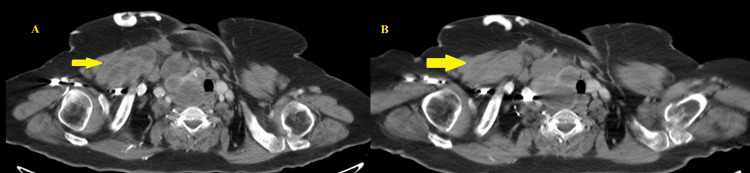
Comparison of CT imaging from May 2025 (A) to August 2025 (B) showing the interval resolution of cystic component in the right pectoralis major muscle CT: computed tomography

The patient was reviewed by the surgical team, but intervention was not pursued due to the lesion's proximity to critical structures (trachea, esophagus, and superior vena cava) and the absence of severe pressure-related symptoms. To address progressive swelling and the risk of compression of adjacent structures, albendazole 400 mg twice daily was initiated (15 mg/kg/day in two divided doses, not exceeding the maximum dose of 800 mg/day). Follow-up imaging with contrast-enhanced CT scan of the neck and chest was performed at 12 weeks post-treatment. This revealed a stable superior mediastinal portion of the hydatid cyst with resolution of the component located within the right pectoralis major muscle, representing partial response toward therapy (Figure 4). Calcified hepatic lesions remained unchanged. She did not report any adverse effects (related to therapy or otherwise), and follow-up complete blood counts and liver function tests remained within range. Considering these findings, she was advised to continue albendazole, with treatment anticipated to extend over several months, guided by clinical and radiological response. Infectious Diseases follow-up was advised at two-monthly intervals.

## Discussion

CE remains a significant zoonotic infection in endemic regions such as South Asia, including Pakistan. The Food and Agriculture Organization of the United Nations (FAO) and the World Health Organization (WHO) ranked* E. granulosus *second among foodborne parasites of global public health importance in 2012 [10]. 

The disease most commonly affects the liver and lungs, while involvement of the head, neck, and mediastinum is exceptional [3-7]. In our patient, the hydatid cyst was located in the right infraclavicular region with extension into the superior mediastinum and contiguous sternoclavicular joint destruction, unusual for both anatomical location and associated bony erosion. A published series showed that only 24 out of 1,056 cases of hydatidosis (2.3%) were localized in the soft tissue [3]. Most reported cases in this anatomical location represent secondary dissemination from a hepatic or pulmonary focus [2]. Previous hepatic and thyroid surgery in this patient is suggestive of primary hydatid disease with secondary seeding occurring hematogenously or intraoperatively, supported by calcified hepatic lesions on imaging. Unfortunately, the absence of prior surgical and diagnostic records limits the confirmation of this interpretation. 

The diagnosis of hydatid disease in atypical sites is challenging, as other differentials (branchial cyst, cold abscess, or neoplasm of thyroid or soft tissue origin) are often prioritized. In this patient, the mass was initially suspected to be a thyroidal malignancy; thus, FNA biopsy was performed. This was inconclusive, emphasizing its limitation in the diagnosis of CE due to the patchy distribution of diagnostic elements like protoscolices or laminated membranes, as well as the associated risk of cyst rupture and anaphylaxis [3]. 

Histopathological examination of hydatid cysts typically reveals an acellular laminated cyst wall, protoscolices with multiple hooklets (observed in less than 50% of cases), and a calcified cyst wall [11]. Other findings include peri-cystic fibrosis, inflammatory infiltrates (eosinophils, plasma cells, lymphocytes, and histiocytes), and a foreign body-type reaction, consisting of fibrous or granulation tissue in the surrounding host tissue [11]. Serologic testing can complement radiologic findings in the diagnosis of CE. The immune response varies according to cyst location, with hepatic cysts eliciting stronger reactions than pulmonary cysts. These assays are also influenced by cyst integrity; intact, unruptured cysts may yield weak or undetectable antibody responses, whereas ruptured cysts typically provoke a marked serologic reaction. Monitoring of titers to assess treatment response is generally considered unreliable [12]. 

Management depends on cyst location, size, and complications. Surgical excision remains the treatment of choice for accessible cysts, aiming for complete removal without rupture [13]. Cysts closely associated with critical structures, multiple cysts, or those with bony involvement may be unamenable to surgery and carry a high risk of morbidity. Hydatid disease of the bone is less sensitive to albendazole than cysts at other sites, requiring higher dosage and long-term administration (years) [14].

Medical management with benzimidazole derivatives such as albendazole or mebendazole is preferred in these scenarios [14]. Generally, small (<7 mm), isolated cysts with minimal surrounding adventitial reaction respond better to treatment than multiloculated or calcified cysts or those with daughter cysts [12]. After 12 weeks of albendazole therapy, our patient demonstrated partial radiological resolution despite unfavorable prognostic features, supporting medical management in cases where surgical intervention is not feasible, but treatment remains indicated.

## Conclusions

This case is unique as it highlights the importance of considering CE in the differential diagnosis of cystic masses in patients from endemic regions, even when anatomical involvement is atypical and there is no known animal exposure. Multidisciplinary management is crucial to ensure optimal outcomes and minimize complications. Finally, inoperable cysts requiring intervention may respond to medical therapy alone, representing an effective alternative approach to management. 
